# Supplementary material for: MetaRibo-Seq measures translation in microbiomes
Source: Nat Commun. 2020 Jun 29;11:3268. doi: 10.1038/s41467-020-17081-z (PMC7324362; doi:10.1038/s41467-020-17081-z)
Supplement: Supplementary file 10 — Supplementary Data 7 [file 41467_2020_17081_MOESM10_ESM.zip › File2/Confidence_High_Taxonomy/422271_out.krona.html]

Javascript must be enabled to view this page.

members
magnitude
magnitudeUnassigned
count
unassigned
taxon
rank

422271\_out

11

2
superkingdom
11

phylum
11
1239

186801
11
class

11
order
186802

31979
family
11

1485
11
genus

1896978
11

SRS016335\_contig\_number\_39595SRS024388\_contig\_number\_9922SRS045826\_contig\_number\_20629SRS047014\_contig\_number\_34180SRS053573\_contig\_number\_contig-100\_25349.25349SRS101433\_contig\_number\_contig-100\_24593.65256SRS1041132\_contig\_number\_contig-100\_3997.40422SRS1041137\_contig\_number\_contig-100\_5323.132902SRS142890\_contig\_number\_contig-100\_36961.90893SRS144506\_contig\_number\_contig-100\_4550.236613SRS144753\_contig\_number\_contig-100\_2053.2054
species
